# Excess Folic Acid Supplementation Before and During Pregnancy and Lactation Activates *Fos* Gene Expression and Alters Behaviors in Male Mouse Offspring

**DOI:** 10.3389/fnins.2019.00313

**Published:** 2019-04-05

**Authors:** Dandan Chu, Longfei Li, Yanli Jiang, Jianxin Tan, Jie Ji, Yongli Zhang, Nana Jin, Fei Liu

**Affiliations:** ^1^Key Laboratory of Neuroregeneration of Jiangsu and Ministry of Education, Co-innovation Center of Neuroregeneration, Nantong University, Nantong, China; ^2^Department of Neurochemistry, Inge Grundke-Iqbal Research Floor, New York State Institute for Basic Research in Developmental Disabilities, New York, NY, United States

**Keywords:** folic acid, pregnancy, lactation, RNA sequencing, *Fos*, behavior

## Abstract

Periconceptional folic acid (FA) supplementation is recommended to prevent neural tube defects and other birth defects. After 20 years mandate food fortification with FA, serum concentration of folate and unmetabolized FA increased significantly in the North American population. But whether excess FA intake impairs neurodevelopment and behavior is still controversial. Here, we treated mice with approximately 2.5-fold (moderate dose of FA, MFA) or 10-fold (high dose of FA, HFA) the dietary requirement of FA 1 week before mating and throughout pregnancy and lactation, and examined behaviors in adult male offspring using open field test, three-chamber sociability and social novelty test, elevated plus maze, rotarod and Morris water maze. We found that early life MFA supplementation increased long-term body weight gain in adults, elevated anxiety-like behavior, and impaired social preference, motor learning and spatial learning ability without modifying motor ability and spatial memory. In contrast, HFA supplementation only induced mild behavioral abnormality. RNA sequencing revealed that FA supplementation altered the expression of brain genes at weaning, among which *Fos* and related genes were significantly up-regulated in MFA mice compared with control and HFA mice. Quantitative real time-PCR (qRT-PCR) and western blots confirmed the increase of these genes. Our results suggested that FA supplementation during early life stage affected gene expression in weaning mice, and exhibited long-term impairments in adult behaviors in a dose-sensitive manner.

## Introduction

Folate is essential for nucleic acid synthesis and methylation reactions as a methyl donor in one-carbon metabolism (Anderson et al., 2012; Crider et al., 2012). Periconceptional folate deficiency increases the risk of congenital malformations, especially neural tube defects (NTDs) (Safi et al., 2012). Overwhelming evidence indicates that 0.4 mg per day of folic acid (FA), the synthetic oxidized form of natural folate, helps protect against NTD and other birth defects. Women planning to get pregnant are recommended to consume FA supplementation from at least 1 month before conception through early pregnancy (Centers for Disease Control, 1992). In addition, FA food fortification was mandated to decrease the incidence of NTDs in the United States, Canada, Chile and other countries since the late 1990s (Ray, 2004; Crider et al., 2011). Due to the FA supplementation and fortification, high concentrations of folate and unmetabolized FA (UMFA) are detected in most maternal and fetal circulation (Kelly et al., 1997; Obeid et al., 2010; Pfeiffer et al., 2012; Plumptre et al., 2015) as well as in breast milk (Page et al., 2017). Meanwhile, potential adverse effects of excess synthetic FA intake have begun to emerge.

It has been reported that high FA intake may mask vitamin B-12 deficiency (Hirsch et al., 2002; Paul and Selhub, 2016), increase the incidence of twin births (Czeizel and Vargha, 2004), accelerate cancer progression (Boyles et al., 2016), suppress thyroid functions during adolescence with deficits in motivation and spatial memory (Sittig et al., 2012), be associated with increased risks of asthma (Whitrow et al., 2009), insulin resistance (Yajnik et al., 2008), and cardiovascular diseases in the offspring (Khot et al., 2015). Although controversial, recent reports also suggest that excess folate intake might be associated with the increasing risk for autism spectrum disorder (ASD) (Choi et al., 2014; Neggers, 2014). Additionally, high supplementation of FA (4 mg/kg or 20 mg/kg diet) during gestation and pregnancy dysregulated gene expression in the cerebellum of neonatal pups (Barua et al., 2014, 2015), and lead to increased ultrasonic vocalizations, greater anxiety-like behavior and hyperactivity (Barua et al., 2014). FA supplementation at 40 mg/kg diet prior to conception exhibited reversal learning impairments in the offspring (Henzel et al., 2017). However, whether gestational and lactational exposure to excess FA has long-term impairments on behaviors and brain gene expression of the adult offspring remain unclear.

In the present study, we treated mice with approximately 2.5-fold (moderate dose of FA, MFA) or 10-fold (high dose of FA, HFA) the dietary requirement of FA prior to and during pregnancy and lactation, compared brain transcriptome between each group in weaning and adult male offspring, and evaluated the behaviors of the adult offspring. We found that MFA but not HFA activated *Fos* and related gene expression, and induced more behavioral abnormalities in adult male offspring, suggesting a dose-sensitive effect of FA supplementation on brain gene expression and long-term modification of behaviors.

## Materials and Methods

### Animals and Treatment

All animal experiments were performed in accordance with National Institutes of Health Guidelines for animal research. ICR mice were initially obtained from SLRC Laboratory Animal Company (Shanghai, China) and bred in the university animal colony. Mice were housed 2–6 per cage on a 12-h light /dark cycle with *ad libitum* access to water and food. All mice were fed NIH-31 open formula diet containing 2 mg FA per kg diet (Xietong Pharmaceutical Biotechnology Co., Ltd., Jiangsu, China), which is generally recommended as the basal dietary requirement for mice (Reeves, 1997). At 8 weeks of age, 6 male (2 male per cage) and 12 female (4 female per cage) mice were randomly divided into three groups and given with de-ionized water (Qipshidze et al., 2011; Mochizuki et al., 2016) containing no FA (control), 3.75 mg/L FA (F7876, Sigma-Aldrich, St. Louis, MO, United States) or 22.5 mg/L FA for 1 week. Then 2 females were caged with 1 male and allowed to mate naturally. FA supplementation lasted throughout the mating period, pregnancy and lactation. Breeding mice consume approximately 5 g diet per mouse daily (Huerkamp and Dowdy, 2008), from which the intake of FA is 10 μg per day. The mean daily consumption of water for an adult mouse is 4 – 8 mL (Bachmanov et al., 2002). Thus, 3.75 mg/L FA (MFA) in the drinking water supplies at least 15 μg FA per day, making the total FA intake from water and food more than 2.5 times the normal dietary intake. Similarly, 22.5 mg/L FA (HFA) supplies at least 90 μg extra intake of FA from water, leading to 10 times the normal dietary intake or more. MFA level was chosen because the total folate intake (400–800 μg/day) from FA fortification and multivitamin were more than twice the recommended dietary allowance (RDA) of dietary folate (100–200 μg/day) in North American populations (Kim, 2008) and the serum folate concentrations increased approximately 2.5-fold postfortification in the U.S. population (Pfeiffer et al., 2012). HFA level was selected because women who have had a child with NTDs are recommended to consume 10 times (4 mg/day) the normal dose for pregnant women (400 μg/day)(Institute of Medicine, 1998).

Pups from all three groups were weaned to normal chow and de-ionized water at postnatal day 21. Male weaning pups (*n* = 5 per group) were sacrificed and the cerebrums were quickly dissected, kept in the ice-cold phosphate-buffered saline (PBS) for 5 min (Wang et al., 2015), snap frozen in liquid nitrogen and stored at −80°C until analysis. Male offspring were weighed at postnatal day 21, 30, 60, 90, 120, and 150 (Con, *n* = 15; MFA, *n* = 16; HFA, *n* = 14). At 5 months of age, male offspring were sacrificed and the brains were quickly dissected (Con, *n* = 16; MFA, *n* = 17; HFA, *n* = 17), weighed in ice-cold PBS, and were kept in the ice-cold PBS for 5 min (Wang et al., 2015). Cerebrums were then dissected, snap frozen in liquid nitrogen and stored at −80°C until analysis.

### Behavioral Tests

All experiments were conducted during the light phase between 8 am and 5 pm. Male offspring from 4 litters per group (2–8 males from each litter) were used. All behavior was recorded by ANYmaze video tracking system (Stoelting Co., Wood Dale, IL, United States) except for Rotarod. Behavioral tests began at the age of 2 months, and were run in the following order: nest formation (Supplementary Methods), open field, sociability and social novelty, marble burying test (Supplementary Methods), elevated plus-maze, rotarod and Morris water maze. The apparatuses were cleaned with water and 75% ethanol, and dried with paper towel between mice.

#### Open Field

Mice were placed in a 48 × 48 cm black plastic arena with 40 cm high walls (Xinruan, Shanghai, China) for a 10-min test session. A 15 × 15 cm square at the center of the apparatus was defined as the center zone. The general exploration and locomotor activity were recorded. The time spent exploring the center zone and self-grooming were recorded as measures of anxiety (Yin et al., 2017) and repetitive behavior, respectively (Kalueff et al., 2016). Con, *n* = 16; MFA, *n* = 16; HFA, *n* = 15.

#### Sociability and Social Novelty

Adult sociability and social novelty were measured by the three-chamber test (Moy et al., 2004) (Ugo Basile, Gemonio, Italy). The apparatus was a rectangular three-chambered box made from 5 mm thick clear Plexiglas. Each chamber was 40 cm L × 20 cm W × 22 cm H, and was separated by clear Plexiglas walls with a central door of 5 cm W × 8 cm H. The social targets were enclosed in round wire cages, which were 15 cm in height, with the diameter of 7 cm and bars spaced 1 cm apart, allowing olfactory and minimal tactile contacts between the bars. The test mouse was first placed into the central chamber of the apparatus and allowed to explore freely for 5 min. After the habituation phase, a stranger mouse was enclosed in a wire cage and placed in the left chamber. An identical empty cage was placed in the right chamber. Then the two side doors were simultaneously lifted, the subject mouse was allowed to explore the entire apparatus for 10 min to quantify social approach behavior. At the end of the sociability phase, the subject mouse was confined to the central chamber with two side doors closed. Another stranger mouse was enclosed in the wire cage that had been empty during the sociability phase. The doors were open again and the subject mouse was allowed to explore the apparatus for 10 min to quantify preference for social novelty. The amount of time spent in each chamber and the time sniffing the stranger mice or the empty cage were automatically recorded. The stranger mice were adult male ICR mice matched to the subjects by age. Con, *n* = 16; MFA, *n* = 18; HFA, *n* = 17.

#### Elevated Plus-Maze

The elevated plus-maze consisted of two open arms (30 × 5 cm) and two enclosed arms (30 × 5 × 20 cm) connected by an open central zone (5 × 5 cm), arranged so that the arms of the same type were opposite each other (Xinruan, Shanghai, China). The apparatus was elevated to a height of 80 cm above the floor, with two open and two enclosed arms opposite facing each other. A mouse was placed in the central zone with its head directed toward a closed arm, and allowed to explore the maze for 5 min. The number of entries into different types of arms and amount of time spent in arms were recorded to evaluate anxiety-like behavior of the animal. Con, *n* = 16; MFA, *n* = 18; HFA, *n* = 16.

#### Accelerating Rotarod

The mice were tested on an accelerating rotarod (Ugo Basile, Gemonio, Italy) for two sessions (Yin et al., 2017). Trials were repeated 3 times each session for a maximum of 500 sec per trial. The cylinder accelerated steadily from 4 to 40 rpm over 2 min. The latency to fall off the apparatus was recorded. The mice were allowed to rest for at least 30 min between trials and 3 h between sessions. Con, *n* = 16; MFA, *n* = 18; HFA, *n* = 17.

#### Morris Water Maze

The test was performed in a black circular pool (180 cm in diameter, 60 cm high) filled with water (23 °C ± 2) made opaque by adding non-toxic black food coloring. The maze was theoretically divided into four equal quadrants. The escape platform (13 cm in diameter) submersed 1 cm below the water surface was placed in the center of one quadrant and kept in the same position during all trials. Training trials were 90 s in duration. The mouse that did not find the platform within 90 s was gently guided toward it. At the end of each trial, the mouse was placed on the platform for 20 s. Four trials per day were given for four consecutive days. After each trial, the mouse was dried in a towel and put back in its home cages. The interval between trials was more than 30 min. One day after the last day of training, the mouse was subjected to a probe trial for 90 s in the absence of the platform. The escape latency in each training trial, swim path, swim distance, swimming speed, the first latency to the platform zone, and the number of times mice crosses the former location of the platform in the probe trail were recorded as measures for spatial learning and memory. Con, *n* = 16; MFA, *n* = 18; HFA, *n* = 17.

### RNA Sequencing for P21d Mice Brain

RNA sequencing was performed by Vazyme Biotech Co., Ltd (Nanjing, China). In brief, total RNA was isolated from the cerebrum using Trizol (Invitrogen, Carlsbad, CA, United States) according to the manufacturer’s protocol. P21d mice brains (*n* = 4 per group) were obtained from 2 litters (2 males from each litter) with different fathers. RNeasy spin columns (Qiagen, Valencia, CA, United States) were used to clean up RNA and remove DNA contaminants. The purity and concentration of isolated RNA were evaluated with NanoDrop^®^ spectrophotometers (Thermo Fisher, Waltham, MA, United States) and Qubit^®^ RNA Assay Kit in Qubit^®^ 3.0 Flurometer (Life Technologies, Carlsbad, CA, United States), respectively. RNA integrity was assessed using the LabChip GX system (Caliper, Newton, MA, United States). RNA Quality Score (RQS) ranges between 7.6 and 8.4. The sequencing libraries were prepared using the VAHTS mRNA-seq v2 Library Prep Kit for Illumina^®^ (Vazyme, Nanjing, China) following manufacturer’s recommendations, and sequenced on an Illumina Hiseq^TM^ X Ten platform (Illumina, San Diego, CA, United States) with 150 bp paired-end reads.

The raw reads were subjected to quality control to remove reads containing adapter, ploy-N and low quality reads. Clean reads were aligned to the mouse mm10 reference genome using TopHat (v2.1.1) and assembled using Cufflinks (v2.2.1) with a reference-based approach. Cuffdiff (v1.3.0) was then used to calculate the number of fragments per kilobase of transcript sequence per millions base pairs mapped (FPKM) for coding genes in each sample. Differential expression was determined by comparing FPKM values of the control samples to those of MFA or HFA samples. Transcripts with corrected *p* < 0.05 and fold change > 2 (the absolute value of log2 ratio > 1) were considered as significantly differentially expressed. Gene ontology (GO) enrichment analysis of differentially expressed genes was implemented with GO::TermFinder. Gene interaction network was implemented based on StringDB database.

### Quantitative Real Time-PCR (qRT-PCR)

Cerebral RNA (*n* = 4 per group) were collected from 2 litters (2 males from each litter) with different fathers. Equal amount of RNA was reverse transcribed to cDNA using HiScript II Q RT SuperMix for qPCR (+gDNA wiper) (R223-01, Vazyme, Nanjing, China). Real-time PCR was performed with SYBR Green Universal Mix (Sigma-Aldrich, St. Louis, MO, United States) on a LightCycler^®^ 480 (Roche Diagnostics, Basel, Switzerland). The PCR conditions were as follows: 95 °C for 10 min followed by 45 cycles of 95°C for 10 s and 60°C for 30 s, and then 95°C for 10 s, 65°C for 60 s, 97°C for 1 s and 37°C for 30 s. Relative levels of mRNA were quantified by the comparative 2^−ΔΔCt^ method and normalized to GAPDH level. Primer sequences are listed in Supplementary Table S7.

### Western Blot

Mice cerebrums (*n* = 5 per group) were collected from 2 litters (2–3 males from each litter) with different fathers and homogenized in 9-fold volume of ice-cold brain lysis buffer (50 mM Tris-HCl, pH 7.4, 8.5% sucrose, 10 mM β-mercaptoethanol, and 2.0 mM EDTA) with protease inhibitor cocktail (Roche, Basel, Switzerland), added 2 × Laemmli sample buffer (250 mM Tris–HCl, pH 6.8, 4% SDS, 20% glycerol, 20% β-mercaptoethanol, 0.008% bromphenol blue), and boiled for 10 min. The protein concentration was determined by Pierce^TM^ 660 nm protein assay kit (Thermo Fisher Scientific, Waltham, MA, United States). Same amounts of protein were separated by sodium dodecyl sulfate-polyacrylamide gel electrophoresis (SDS-PAGE), and were electro-blotted on polyvinylidene difluoride (PVDF) membrane. After blocking with 5% fat-free milk in TBS buffer (50 mM Tris-HCl, pH 7.4, 150 mM NaCl) for 30 min, the membrane was incubated with rabbit anti-Fos antibody (1:500, 26192-1-AP, Proteintech, Rosemont, IL, United States) overnight at room temperature. After washing three times with TBST (0.05% Tween-20 in TBS), the membrane was incubated with peroxidase affinipure goat anti-rabbit IgG (111-035-144, Jackson ImmunoResearch, West Grove, PA, United States) for 2 h at room temperature. After three washes in TBST, the membrane was incubated with Enhanced chemiluminescence (ECL) kit (Thermo Fisher Scientific, Rockford, IL, United States), and exposed to X-ray film (Kodak, Rochester, NY, United States). Band intensities were quantified using Multi Gauge software (Fujifilm, Kanagawa Prefecture, Japan).

### Statistical Analysis

Data were presented as mean ± standard error of the mean (SEM). The statistical significance was analyzed by one-way or two-way analysis of variance (ANOVA) followed by Bonferroni’s *post hoc* test for multiple comparisons using GraphPad Prism 6 (GraphPad Software Inc., San Diego, CA, United States). The body weights were analyzed using two-way repeated measures ANOVA. *p* < 0.05 was considered statistically significant.

## Results

### FA Supplementation During Pregnancy and Lactation Affects Male Offspring Growth

**FIGURE 1:**
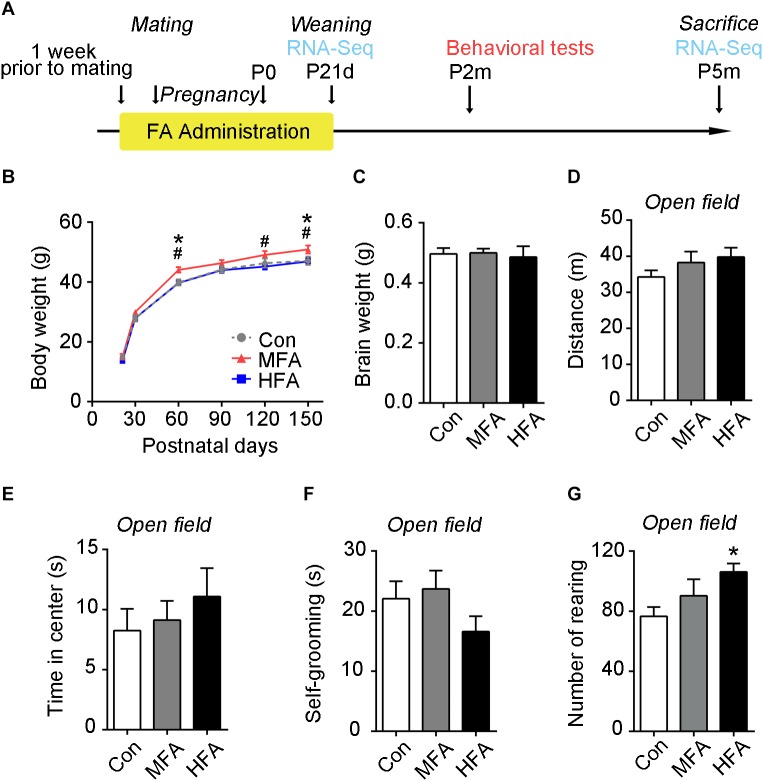
Effects of FA supplementation on general growth and performance in the open field of the male offspring. **(A)** Flowchart of the study design. **(B)** Mean body weight from weaning to postnatal day 150 (*n* = 14–16). **(C)** Mean brain weight at postnatal day 150 (*n* = 16–17). **(D–G)** Mice were subjected to an open field test, and the distance traveled in the arena **(D)**, the time spent in the central square **(E)**, the time spent self-grooming **(F)**, and the number of rearing **(G)** were measured (*n* = 15–16). ^∗^, #, *p* < 0.05. ^∗^, compared to control. #, compared to HFA.

To investigate whether excess FA exposure before and during pregnancy and lactation affected physical growth of the offspring, the mice were supplied with 3.75 mg/L (approximately 2.5 times the dietary requirement, MFA hereafter) or 22.5 mg/L (approximately 10 times the dietary requirement, HFA hereafter) FA in their drinking water from 1 week prior to mating through postnatal day 21 (Figure 1A). After weaning, offspring were fed with normal tap water. We tracked the body weight of the offspring for 5 months. No difference in weaning weight was found. However, MFA mice gained more body weight than the control and HFA mice later in life, especially at postnatal day 60 and 150 (Figure 1B, main effect of time, *F*_5,205_ = 2226, *p* < 0.0001; significant FA × time interaction: *F*_10,205_ = 3.045, *p* = 0.0013), indicating that MFA supplementation during early life could influence physical growth in adult male offspring. Brain weights of the FA-supplied groups were not changed at 5 months of age (Figure 1C, *F*_2,47_ = 1.452, *p* = 0.2444).

### FA Supplementation Modifies Behaviors in Male Offspring

To evaluate behaviors of the FA-exposed offspring, we tested the adult male offspring in a series of behavioral tests. The open field test was used to evaluate locomotor activity and emotional behavior. The overall distance traveled (Figure 1D, *F*_2,44_ = 16.274, *p* = 0.2898) in the open field was unchanged in the MFA and HFA groups, indicating that general motor ability was not affected by FA supplementation. The HFA mice exhibited a greater incidence of rearing, showing more exploratory activity (Figure 1G, *F*_2,44_ = 3.351, *p* = 0.0442). No significant difference was found in the time spent in the central square (Figure 1E, *F*_2,44_ = 0.5540, *p* = 0.5786), self-grooming (Figure 1F, *F*_2,44_ = 1.648, *p* = 0.2041) and latency to first enter the central zone (Supplementary Figure S1A) among groups.

**FIGURE 2:**
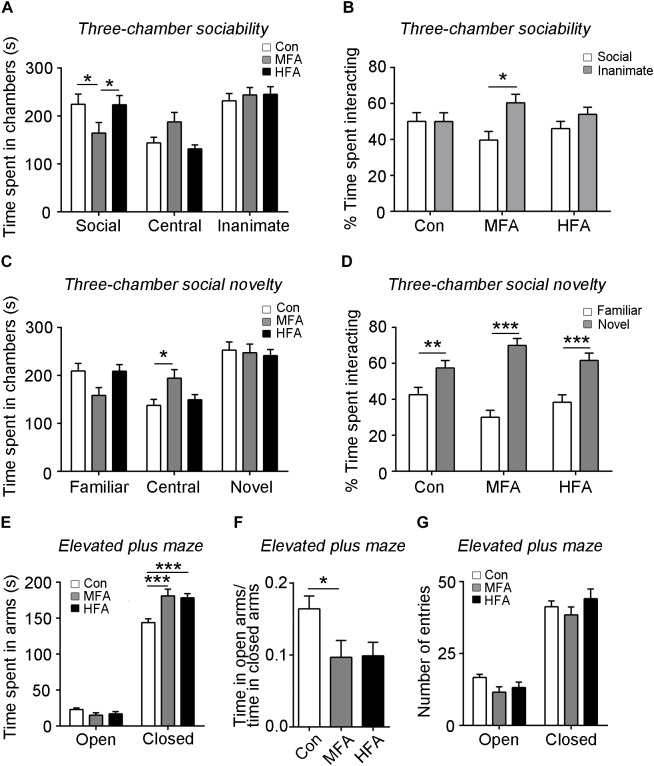
FA supplementation altered offspring sociability and induced anxiety-like behavior. **(A–D)** The three-chamber sociability and social novelty test (*n* = 16–18). The time spent in chambers in the sociability phase **(A)** and the social novelty phase **(C)**, the percentage of time spent interacting with social and inanimate targets **(B)** in the sociability phase, and the percentage of time spent interacting with familiar and novel social targets **(D)** in the social novelty phase were recorded. **(E–G)** Mice were tested in the elevated plus maze. The time spent **(E)** and the number of entries **(G)** into arms were recorded (*n* = 16–18). **(F)** The ratio of time spent in the open arms to the time spent in the closed arms. ^∗^*p* < 0.05, ^∗∗^*p* < 0.01, ^∗∗∗^*p* < 0.001.

The three-chamber sociability and social novelty test were used to measure social behavior in the FA-exposed mice. During the sociability phase, the MFA group spent significantly less time in the chamber containing a stranger mouse (social target) than did the control and HFA group (Figure 2A, main effect of chamber: *F*_2,144_ = 18.74, *p* < 0.0001; significant FA × chamber interaction: *F*_4,144_ = 3.649, *p* = 0.0073), and spent less time sniffing the social target than the inanimate target (Figure 2B, main effect of target, *F*_1,96_ = 6.480, *p* = 0.0125). The HFA group behaved normally in the sociability phase (Figure 2A,B). In the social novelty phase, the time spent in the chamber containing the familiar mouse (familiar target) and a new unfamiliar mouse (novel target) were not affected in both MFA and HFA groups (Figure 2C, main effect of chamber: *F*_2,144_ = 24.81, *p* < 0.0001; significant FA × chamber interaction: *F*_4,144_ = 3.937, *p* = 0.0046). However, MFA mice spent more time in the central chamber, as compared to the control, which might reflect low levels of exploratory and increased anxiety-like behaviors (Moy et al., 2004). All the three groups of mice spent more time exploring the novel target than the familiar target (Figure 2D, main effect of target: *F*_1,96_ = 76.70, *p* < 0.0001; significant FA × target interaction: *F*_2,96_ = 6.615, *p* = 0.0020), without any difference in the preference for social novelty. These results suggested that MFA supplementation decreased social activity without changing the preference for social novelty, while HFA exposure had no effect on the social behavior of the offspring.

The elevated plus-maze was conducted to assess anxiety-like behavior in the mice. Mice of the MFA and HFA group spent more time in the closed arms compared to the control mice (Figure 2E, main effect of arm: *F*_1,94_ = 1068, *p* < 0.0001; main effect of FA: *F*_2,94_ = 4.354, *p* = 0.0156; significant FA × arm interaction: *F*_2,94_ = 9.684, *p* = 0.0001). The ratio of the time spent in the open arms to that in closed arms was significantly decreased in MFA mice, showing elevated anxiety-like behavior (Figure 2F, *F*_2,47_ = 3.449, *p* = 0.0400). The numbers of entries into each arm were not different among groups (Figure 2G, main effect of arm: *F*_1,47_ = 280.5, *p* < 0.0001).

**FIGURE 3:**
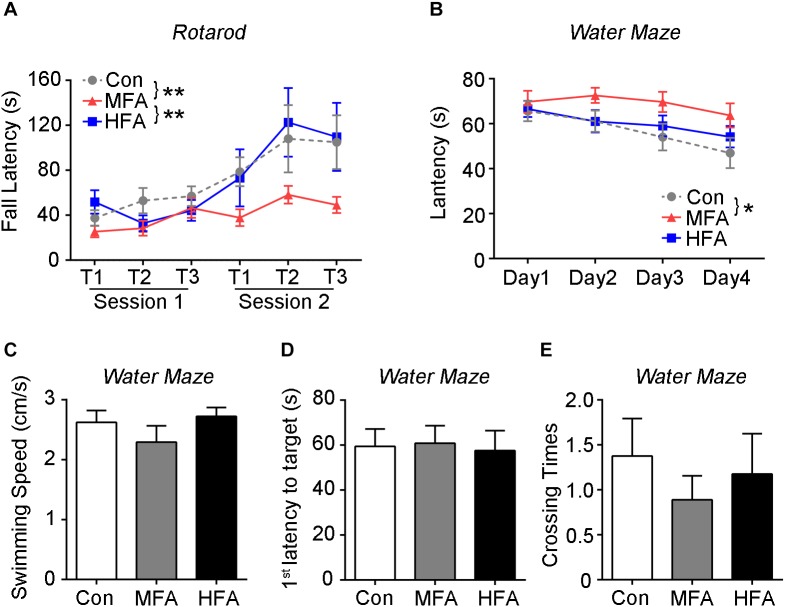
FA supplementation impaired motor and spatial learning in the offspring. **(A)** Motor behavior of the mice was assessed by the accelerating rotarod (*n* = 16–18). **(B–E)** The Morris water maze test is used to assess spatial learning and memory (*n* = 16–18). During the training phase, **(B)** the time traveled to reach the platform (latency) were analyzed. Probe trials were performed 1 day after the last training trial. The swimming speed **(C)**, the first latency to reach the platform zone **(D)**, and the crossing number of the platform zone **(E)** were analyzed. ^∗^*p* < 0.05, ^∗∗^ *p* < 0.01.

The mice were then assessed for motor learning and coordination on an accelerating rotarod. The three groups showed similar coordination during the initial trials. Subsequently, both the control and HFA group exhibited an increase in the latency to fall from the rotarod, whereas the MFA group displayed no significant improvements in their performance (Figure 3A, main effect of trail: *F*_5,288_ = 7.132, *p* < 0.0001; main effect of FA: *F*_2,288_ = 7.894, *p* = 0.0005), suggesting impaired motor learning in MFA-administrated offspring.

Morris water maze test was used to evaluate spatial learning and memory of FA-exposed offspring. During the training days, MFA mice spent more time than the control before reaching the platform (Figure 3B, main effect of day; *F*_3,144_ = 4.334, *p* = 0.0059; main effect of FA; *F*_2,48_ = 4.495, *p* = 0.0162), displaying delay in spatial learning. However, in the probe trial, the swimming speed, the time spent to reach the target, and the times mice swam across the target zone were not different among groups (Figure 3C–E, *F*_2,48_ = 1.138, *p* = 0.3290; *F*_2,48_ = 0.04188, *p* = 0.9590; *F*_2,48_ = 0.4151, *p* = 0.6626), indicating normal motor ability and spatial memory of the FA-treated mice.

Taken together, these results suggested that FA supplementation did not affect athletic ability of the male offspring, although MFA supplementation did lead to more weight gain with age. MFA but not HFA elevated anxiety-like behavior, impaired social preference, motor learning and spatial learning in the male offspring.

### FA Supplementation Alters Gene Expression in Male Offspring at Weaning

To determine whether excess FA supplementation during the early stages of life influenced brain transcriptome, we collected the mice cerebrums at weaning (postnatal day 21, P21d), and subjected them to RNA sequencing. We identified 176 differential expression genes in MFA brains compared with the control (103 up-regulated, 73 down-regulated, Supplementary Table S1), and only 96 differential expression genes in HFA brains (34 up-regulated, 62 down-regulated, Supplementary Table S2). Comparison between MFA and HFA samples showed the lowest difference, with 59 genes differentially expressed (43 up-regulated, 16 down-regulated, Supplementary Table S3). These results were consistent with the extent of behavioral abnormality seen in adult MFA and HFA mice.

**FIGURE 4:**
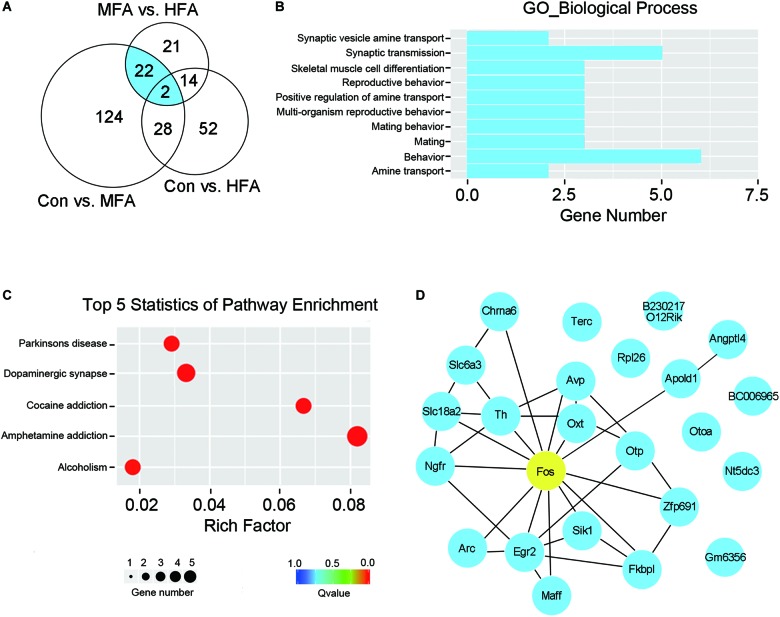
FA supplementation changed brain gene expression at weaning. RNA isolated from the cerebrum was subjected to sequencing (*n* = 4). **(A)** Venn diagram illustrated the number of differentially expressed genes unique or shared between each comparison. Genes differentially expressed exclusively in MFA mice was indicated in blue. **(B)** GO term enrichment analysis for biological process of the genes differentially expressed exclusively in MFA mice. **(C)** Top 5 statistics of Kyoto Encyclopedia of Genes and Genome (KEGG) pathway enrichment for the genes differentially expressed exclusively in MFA mice. **(D)** Gene interaction network representing the 24 genes differentially expressed exclusively in MFA mice. 16 of 24 genes were connected directly or indirectly with *Fos* gene (indicated in yellow).

Since MFA mice exhibited more obvious behavioral phenotypes than HFA mice, we selected 24 genes that are differentially expressed exclusively in MFA but not in HFA brains (Figure 4A and Table 1) and categorized them according to gene ontology (GO) analysis (Figure 4B). The top enriched categories of biological processes were behavior and synaptic transmission. KEGG (Kyoto Encyclopedia of Genes and Genomes) analysis revealed 5 major enrichment pathways of these genes involving amphetamine addiction, dopaminergic synapse, cocaine addiction, Parkinson’s disease and alcoholism (Figure 4C). We then generated a protein interaction network that connected these genes, and found 16 of 24 genes were directly or indirectly associated with the immediate early gene *Fos* (Figure 4D).

**Table 1:** Differentially expressed genes in MFA compared to Control and HFA.

Gene Symbol	Ensembl ID	Fold change		Adjusted *p-value*	
		log_2_(MFA/Con)	log_2_(MFA/HFA)	MFA vs. Con	MFA vs. HFA
Angptl4	ENSMUSG00000002289	1.3085	1.3961	0.0306	0.0379
Apold1	ENSMUSG00000090698	1.6991	1.8414	0.0035	0.0114
Arc	ENSMUSG00000022602	1.0834	1.7816	0.0035	0.0114
Avp	ENSMUSG00000037727	2.1244	3.5785	0.0035	0.0114
B230217O12Rik	ENSMUSG00000097785	1.1536	1.1467	0.0035	0.0328
BC006965	ENSMUSG00000041674	1.5948	2.1452	0.0154	0.0114
Chrna6	ENSMUSG00000031491	1.9037	1.0524	0.0035	0.0379
Egr2	ENSMUSG00000037868	1.2330	2.4399	0.0035	0.0114
Fkbpl	ENSMUSG00000033739	1.2831	1.6290	0.0083	0.0114
Fos	ENSMUSG00000021250	1.1577	1.9900	0.0035	0.0114
Gm6356	ENSMUSG00000091400	−1.7326	−1.6205	0.0035	0.0196
Maff	ENSMUSG00000042622	1.5320	1.4854	0.0103	0.0379
Ngfr	ENSMUSG00000000120	1.1116	1.2031	0.0083	0.0114
Nt5dc3	ENSMUSG00000054027	2.0304	1.1502	0.0035	0.0114
Otoa	ENSMUSG00000034990	3.0462	2.8664	0.0035	0.0114
Otp	ENSMUSG00000021685	1.9204	1.3784	0.0035	0.0114
Oxt	ENSMUSG00000027301	1.1376	1.6930	0.0294	0.0114
Rpl26	ENSMUSG00000060938	3.1681	2.8555	0.0035	0.0114
Sik1	ENSMUSG00000024042	1.1791	1.2564	0.0035	0.0114
Slc18a2	ENSMUSG00000025094	1.2186	1.0122	0.0035	0.0114
Slc6a3	ENSMUSG00000021609	1.8408	1.2427	0.0035	0.0114
Terc	ENSMUSG00000064796	−Infinity	Infinity	0.0456	0.0114
Th	ENSMUSG00000000214	1.2418	1.0694	0.0035	0.0114
Zfp691	ENSMUSG00000045268	1.4209	1.2762	0.0035	0.0196

To assess whether FA supplementation before weaning affected gene transcription in adult brain, we collected the cerebrums of the mice from each group after the behavioral tests (5 months postpartum, P5m), and also subjected them to RNA sequencing. At P5m, HFA brains still had 80 differential expressed genes compared with the control, suggesting that high dose of FA supplementation at early life stage could affect gene expression in adult brain (70 up-regulated, 10 down-regulated, Supplementary Table S5). Strikingly, the number of differential expression genes in MFA brains dramatically decreased at P5m. Only 9 genes (2 up-regulated, 7 down-regulated) were differentially expressed in MFA brains compared with the control, 4 of which were only changed in MFA brains but not in HFA brains (Supplementary Table S4). However, none of these 4 genes are protein-coding genes, and their differential expression could not be confirmed by qPCR (data not shown). Additionally, the transcription of 24 differential expression genes in MFA brains at P21d returned to normal levels at P5m (Supplementary Table S6). Taken together, HFA exposure before weaning induces long-term alterations in brain gene expression, whereas MFA caused more remarkable transient alteration of brain transcriptional profile in weaning mice, which might be responsible for the behavior modification in adulthood.

### *Fos* Gene Expression Is Up-Regulated in MFA Mice Brains

**FIGURE 5:**
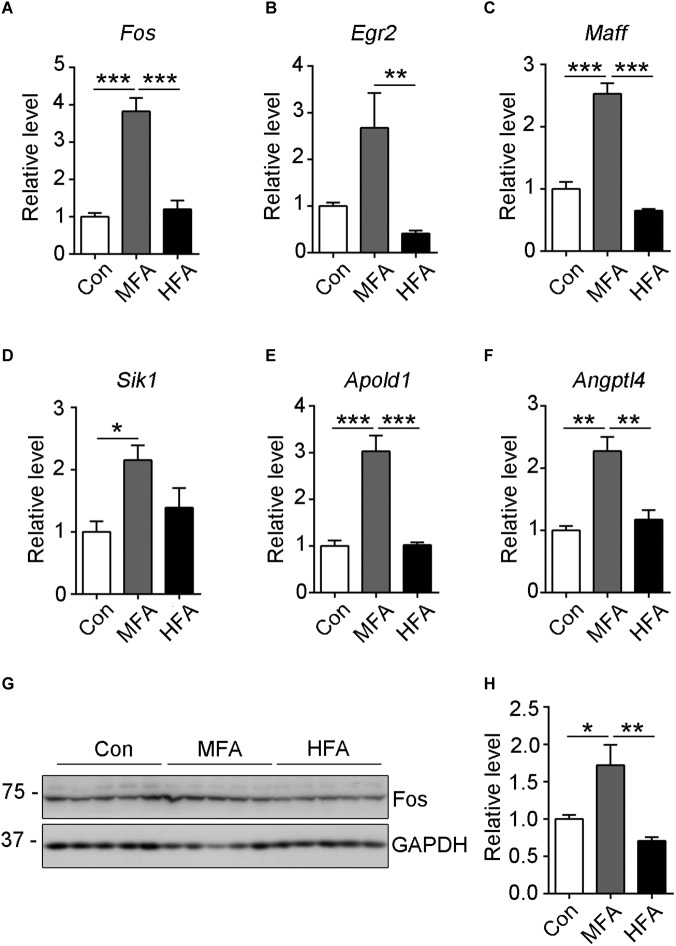
Expression of selected differentially expressed genes as validated by qRT-PCR or western blot. (A–F) The relative mRNA expression level of *Fos* **(A)**, *Egr2* **(B)**, *Maff* **(C)**, *Sik1* **(D)**, *Apold1* **(E)** and *Angptl4* **(F)** genes were determined by qRT-PCR (*n* = 4). GAPDH was used for normalization. **(G)** The c-Fos protein level was analyzed by western blots. **(H)** The relative protein level of c-Fos was calculated after normalizing to GAPDH (*n* = 5). ^∗^*p* < 0.05, ^∗∗^ *p* < 0.01, ^∗∗∗^*p* < 0.001.

The transcription levels of the genes exclusively expressed in MFA brains were further validated by qPCR. Among these genes, *Fos* mRNA level showed the most significant change in MFA brains, with an over 3.5-fold increase as compared with that in either control or HFA brain (Figure 5A, *F*_2,9_ = 38.01, *p* < 0.0001). Besides, *Egr2* (early growth response 2), *Maff* (MAF bZIP transcription factor F), *Sik1* (salt inducible kinase 1), *APOLD1* (apolipoprotein L domain containing 1) and *ANGPTL4* (angiopoietin like 4) mRNA levels had a more than 2-fold increase in MFA brain (Figure 5B–F, *F*_2,9_ = 7.322, *p* = 0.0130; *F*_2,9_ = 70.84, *p* < 0.0001; *F*_2,9_ = 5.550, *p* = 0.0269; *F*_2,9_ = 31.64, *p* < 0.0001; *F*_2,9_ = 17.74, *p* = 0.0008). Then the protein levels of c-Fos were further analyzed by western blot. In MFA brains, the level of c-Fos protein was more than 1.2-fold higher compared with that in the control or HFA brains (Figure 5G,H, *F*_2,12_ = 10.16, *p* = 0.0026). These results showed that the expression of *Fos* gene was increased both trancriptionally and translationally in MFA but not HFA-exposed mice brain at weaning.

## Discussion

Folic acid supplementation and food fortification have long been accepted to prevent NTDs (Centers for Disease Control, 1992), and have already increased the serum concentration of folate 2.5-fold in both male and female population in the United States (Pfeiffer et al., 2012). But the potential influence of excess perigestational and lactational FA intake on the behavior and gene expression in the offspring is not thoroughly understood. Since both paternal and maternal supplementation of FA could transmit adverse effects to the offspring (Ly et al., 2017; Patel and Sobczynska-Malefora, 2017), we fed both the male and female mice with excess FA 1 week prior to mating and through the pregnancy and lactation, and found that MFA increased long-term body weight gain and resulted in abnormal behaviors in the adult male offspring, including impaired social preference, elevated anxiety-like behavior, deficient motor learning and spatial learning ability. MFA altered the expression of brain genes at weaning, among which *Fos* and related genes expression displayed most dramatic fold changes. Surprisingly, although HFA exposure did induce long-lasting impact on brain gene expression, it brought about less differentially expressed genes than MFA did at weaning, and only caused minor behavioral abnormality in elevated plus maze.

Excess of maternal FA supplementation has been reported to increase body weight gain in rodent (O’Neill et al., 2014). Male mouse pups whose mothers were fed FA at 4 mg/kg diet weighed more than the control mice on postnatal Day 4 and Day 6 (Barua et al., 2014). Maternal supplementation of 40 mg FA/kg diet increased body weight gain of female rat offspring (Keating et al., 2015). Excess intake of FA (5-fold above the recommended level) in the gestational diet activated hypothalamic neuropeptide Y (NPY)-positive neurons at birth and pro-opiomelanocortin (POMC)-positive neurons at 9 weeks post-weaning in the arcuate nucleus of male rat offspring, which may contribute to increased food intake and body weight gain later in life (Yang et al., 2018). Consistent with previous reports, MFA mice exhibited long-term body weight gain at 5 months of age. Nevertheless, increased body weight did not affect the motor ability of MFA mice in the behavioral tests.

High FA supplementation is a risk of abnormal behavior in rodents. Pregnant mothers’ diet with extra doses of FA (4 mg/kg diet) induced behavioral deficiency including more ultrasonic vocalization, anxiety, and hyperactivity in mice offspring (Barua et al., 2014). Rats fed about three times the recommended daily allowance of FA (8 mg/kg diet) from 30 to 60 days of age, exhibited both motivation and memory deficits on a spatial water maze task (Sittig et al., 2012). Weaning pups born to mothers fed with 20 mg FA/kg diet had a negative discrimination index in the novel object recognition test (NOR), indicating short-term memory impairment similarly to that observed in *Mthfr^−/−^* mice (Bahous et al., 2017). In contrast, supplementation of 2 mg FA/kg or 20 mg FA/kg diet during pregnancy prevented memory deficits after neonatal hypoxia-ischemia (Deniz et al., 2018). In this study, we tested the adult but not the weaning mice in NOR, but found no significant difference among groups (data not shown). HFA (roughly 10 times the dietary requirements) showed limited deficits in the behaviors of offspring, with even more exploratory activity as indicated by increased rearing in the open field test, while MFA (roughly 2.5 times the dietary requirements) caused more obvious behavioral abnormalities in the adult male offspring, indicating that moderate but not high FA exposure during early life could impair adult mice behaviors. However, the activity of dihydrofolate reductase (DHFR), the enzyme required to convert FA to dihydrofolate and tetrahydrofolate to acquire metabolic activity *in vivo*, is less than 2% in human liver of that in rat liver. Also the variation of DHFR activity is nearly 5-fold among the human samples in contrast to rats (Bailey and Ayling, 2009), implying that human and rodents may have different sensitivity on FA. Therefore, whether comparable dosage of FA exposure could cause behavioral abnormality in children requires further studies.

It is still controversial whether excess FA increases the risk of ASD (Wiens and DeSoto, 2017), a neurodevelopmental disorder characterized by deficits in social interaction, impaired communication, and repetitive behaviors (Liptak et al., 2008). Some studies reported a protective effect of FA supplementation against ASD (Schmidt et al., 2012; Suren et al., 2013; Frye et al., 2018), while others claimed an increased risk (Beard et al., 2011; Desoto and Hitlan, 2012). A recent work suggested a “U shaped” hypothesis – moderate (3–5 times/week) maternal multivitamin supplementation was correlated to a decreased risk of ASD, whereas extremely low or high supplementation was correlated to an increased risk (Raghavan et al., 2018). In the present study, although MFA mice performed poorly in sociability tests, neither of the FA-supplied groups displayed any significant repetitive behaviors as evaluated by self-grooming (Figure 1F) and nest formation (Supplementary Figure S1B). Marble burying tests even showed decreased stereotyped, repetitive behaviors in FA-supplied mice (Supplementary Figure S1C). These results indicated that these two dosages of FA were not sufficient to induce autism-like behaviors in mice.

Exposure to high dose FA (20 mg/kg) diet during gestation interfered gene expression in the cerebellum of postnatal day 1 mouse pups. The dysregulated genes included transcription factors, imprinted genes, neurodevelopmental genes and genes associated with ASD, and the majority of them are down-regulated (Barua et al., 2015). In this study, we found that both moderate and high dose of FA supplementation changed cerebral gene expression profiles in weaning pups. Surprisingly, compared to HFA, MFA drove a 1.8-fold increase in the number of differentially expressed genes. In addition, 59% differentially expressed genes are up-regulated rather than down-regulated in MFA mice, whereas only 35% differentially expressed genes are up-regulated in HFA. Among the 24 genes that are differentially expressed exclusively in MFA brain, 22 genes were up-regulated. These results implying that the influences of MFA on adult mice behaviors might be associated with the activation of cerebral gene expression.

*Fos* and related genes such as *Egr2*, *Maff*, *Sik1*, *Apold1*, and *Angptl4* were significantly up-regulated in MFA mice brain, and most of the differential genes that exclusively expressed in MFA were associated with *Fos*. The immediate early response genes like *Fos* and *Arc* are rapidly activated following exposure to multiple stressors (Kruijer et al., 1984; Müller et al., 1984; Kapolowicz and Thompson, 2016), including handling and maternal separation (Ryabinin et al., 1999; Sanders and Anticevic, 2007). In rodents, increase of Fos mRNA and protein were detectable 15 min after mild stress exposure (Imaki et al., 1992; Viltart et al., 2006). Thus, up-regulated expression of *Fos* and related genes might reflect increased sensitivity to stress in MFA mice. c-Fos is considered as a switch converting short-term stimuli into long-term responses (Velazquez et al., 2015). *Fos* knockout mice presented about 40–60% reduction in their body weight, and showed behavioral alterations such as learning impairment, hyperactivity and abnormal sexual behavior (Velazquez et al., 2015), and reduced cocaine-induced locomotor sensitization (Zhang et al., 2006). *Fos* and *Egr2* have been reported to be activated in germ-free mice that have anxiety-related behavior and impaired social behavior (Stilling et al., 2015). On the other hand, *Egr2*-deficient mice showed improved performance in motor learning in the rotarod, and in object recognition task (Poirier et al., 2007). However, whether activation of *Fos* and related genes at weaning is directly responsible for the long-term body weight gain and behavior changes in MFA-supplied mice requires further investigation.

Folate is an essential source of one carbon group used for DNA methylation (Crider et al., 2012). Methyl supplementation containing folate were considered to increase the levels of DNA methylation and then inhibit certain gene expression (Cooney et al., 2002; Sittig et al., 2012; Barua et al., 2016a,b). Nevertheless, emerging evidence indicates that excessive intake of FA accounts for not only DNA hypermethylation but also hypomethylation (Sie et al., 2011; Schaevitz and Berger-Sweeney, 2012; Cho et al., 2013; Aarabi et al., 2018). In a model of combined spinal cord and peripheral nerve injury, mice treated with 80 μg FA/kg body weight increased global DNA methylation and enhanced neuroregeneration. But lower (20 μg/kg body weight) and higher doses (400 μg/kg and 800 μg/kg body weight) of FA leads to global DNA hypomethylation and impaired axon regeneration (Iskandar et al., 2010). Notably, intraperitoneal injection of FA 800 mg/kg body weight is roughly equal to the supplementation of 4 mg/L FA in drinking water for a mouse weighted 20 g that consumes 4 ml H_2_O/day, which is comparable to 3.75 mg/L FA supplementation (MFA) used in this study. Thus, we speculated that MFA might promote the expression of *Fos* and related genes by modifying DNA hypomethylation. We used bisulfite sequencing PCR (BSP) to determine the patterns of CpG methylation from -1879 to -1505 bp upstream of *Fos* gene transcription start site (TSS), but did not detect any significant changes in genomic DNA methylation in MFA or HFA brains (Supplementary Figure S2). More research is needed to determine whether the methylation status is altered in other regions of *Fos* gene or in regulatory sites of other related genes like *Egr2*. In addition, since FA affects DNA methylation in a dose-sensitive manner, it is possible that 22.5 mg/L FA (HFA) might modulate global DNA methylation and/or single gene methylation pattern less efficiently than MFA, leading to less differentially expressed genes at weaning, and then less impact on adult mice behaviors.

## Conclusion

We found that excess FA supplementation prior to and during pregnancy and lactation changed gene expression profile at weaning, and had long-term behavioral effects in adult male mouse offspring in a dose-sensitive manner. However, the influences of increasing FA intake on human behaviors require more intensive investigation, and the dosage of FA supplementation throughout pregnancy and lactation should be more cautiously evaluated.

## Ethics Statement

This study was carried out in accordance with the recommendations of Guidelines for the Management of Laboratory Animals in Jiangsu Province, laboratory animal ethics committee of Nantong university’. The protocol was approved by the ‘laboratory animal ethics committee of Nantong university.’

## Author Contributions

FL and DC conceived the research. DC, LL, YJ, JT, JJ, YZ, and NJ performed the experiments. DC, LL, and FL analyzed and interpreted the results. DC and FL drafted the manuscript. LL, YJ, and FL critically revised the manuscript.

## Conflict of Interest Statement

The authors declare that the research was conducted in the absence of any commercial or financial relationships that could be construed as a potential conflict of interest.
