# Supplementary material for: Excess Folic Acid Supplementation Before and During Pregnancy and Lactation Activates Fos Gene Expression and Alters Behaviors in Male Mouse Offspring
Source: Front Neurosci. 2019 Apr 5;13:313. doi: 10.3389/fnins.2019.00313 (PMC6460239; doi:10.3389/fnins.2019.00313)
Supplement: Supplementary file 1 [file Data_Sheet_1.doc]

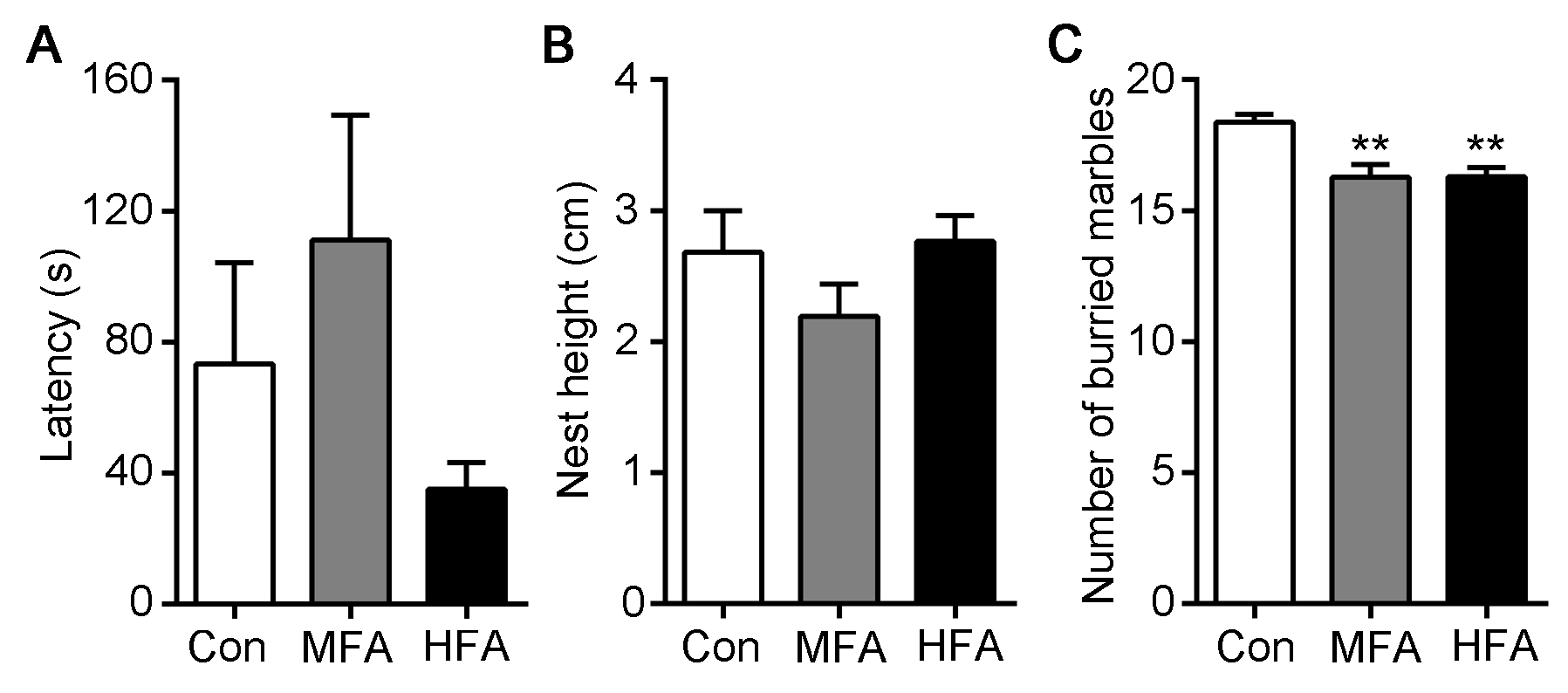


**Supplementary Figure S1.** Effects of FA supplementation on **the latency to enter the center of the open field, nest formation and marble burying in the male offspring. (A)** Latency to first enter the central zone of the open field was not different among groups (*p* = 0.3344, Kruskal-Wallis test). **(B)** The nest height was not significantly changed in MFA and HFA mice. (*F*_2, 48_ = 1.501, *p* = 0.2332, one-way ANOVA). **(C)** MFA and HFA mice buried fewer marbles than the control mice. (*F*_2, 48_ = 9.211, *p* = 0.0004, one-way ANOVA followed by Bonferroni’s *post hoc* test). ** *p*< 0.01. Data were presented as mean ± SEM.


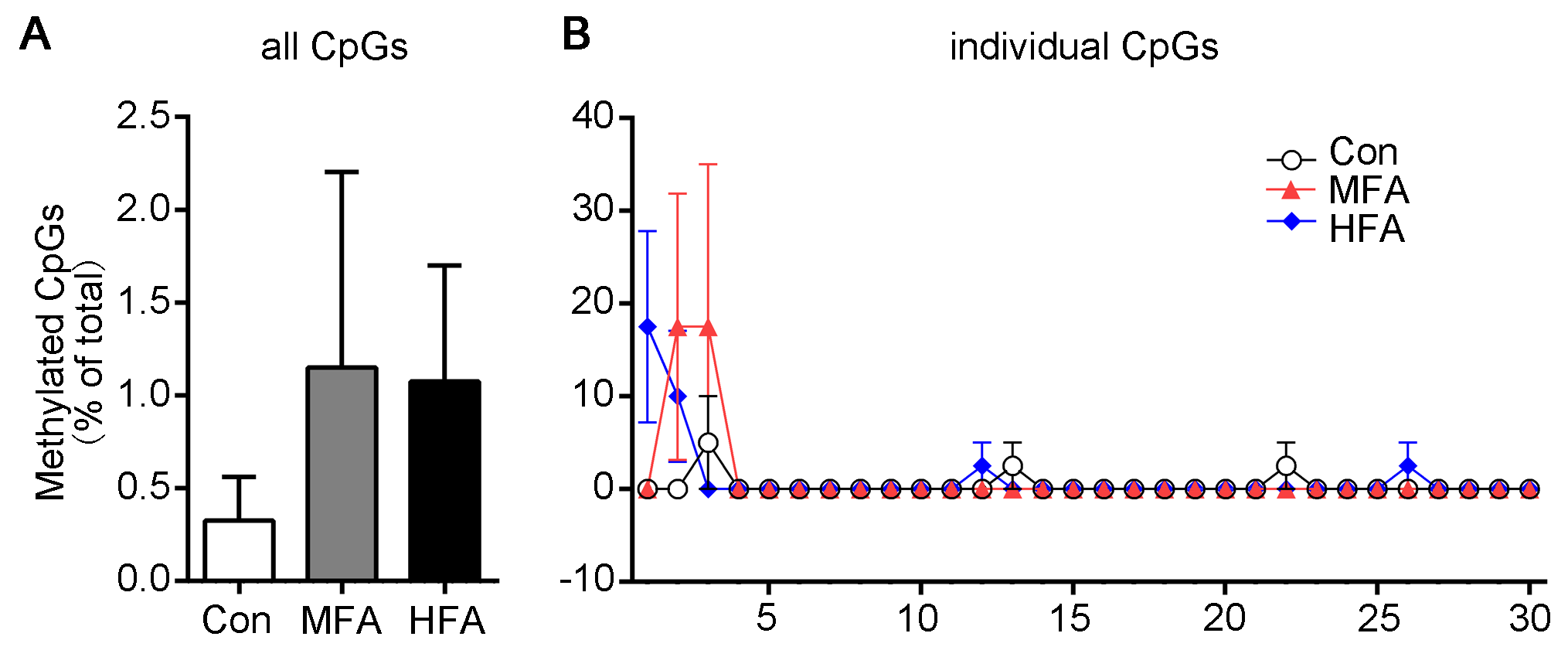


**Supplementary Figure S2. DNA methylation pattern of *Fos* promoter locus were not significantly changed in MFA and HFA mice brain.** Bisulfite sequencing was used to determine the patterns of CpG methylation from -1879 to -1505 bp upstream of *Fos* gene transcription start site. (**A**) The average methylation percentages of all CpG sites were not different among groups (*F*_2, 9_ = 0.4023, *p* = 0.6802, one-way ANOVA). (B) Methylation status of 30 individual CpG sites showed no significantly differences ­­among groups (*F*_58, 270_ = 1.133, *p* = 0.2541, two-way ANOVA). Data were presented as mean ± SEM.

**Supplementary Methods**

**Nest formation**

The test mouse was singly housed in a new cage 30 min before testing. Then, a 5 × 5 cm piece of cotton nesting material was introduced into each cage to permit nesting. After 90 min, the nest height was measured. Con, n = 16; MFA, n = 18; HFA, n = 17.

**Marble burying test**Marble-burying test was applied to assess repetitive-like behaviors in mice. A single mouse was first placed in a clean cage (28 cm L × 18 cm W × 12 cm H) with 4 cm of fresh corncob bedding for a 10-min habituation. Then the mouse was removed and 20 dark green glass marbles (15 mm in diameter) were overlaid on the bedding equidistant in a 4 × 5 arrangement to cover the whole cage surface. The mouse was then returned in the test cage and allowed to bury the marbles for 10 min. At the end of the test, the mouse was removed and the number of marbles buried (﹥75% marble covered by bedding material) was recorded. Con, n = 16; MFA, n = 18; HFA, n = 17.

**RNA sequencing for P5m mice brain**

RNA sequencing for P5m mice (n = 3 per group) was performed by Annoroad Gene Technology Corporation (Beijing, China). Samples were collected from 2 litters (1-2 males from each litter) with different fathers. Total RNA was isolated using phenol/chloroform method. RNA purity was checked using the kaiaoK5500® Spectrophotometer (Kaiao, Beijing, China). RNA integrity and concentration was assessed using the RNA Nano 6000 Assay Kit of the Bioanalyzer 2100 system (Agilent Technologies, CA, USA). RNA integrity number (RIN) ranges between 9.0 and 9.2. A total amount of 2 μg RNA per sample was used as input material for the RNA sample preparations. Sequencing libraries were generated using NEBNext® Ultra™ RNA Library Prep Kit for Illumina® (#E7530L, NEB, USA) following the manufacturer’s recommendations. RNA concentration of library was measured using Qubit® RNA Assay Kit in Qubit® 3.0 to preliminary quantify and then dilute to 1ng/μl. Insert size was assessed using the Agilent Bioanalyzer 2100 system (Agilent Technologies, CA, USA), and qualified insert size was accurate quantification using StepOnePlus™ Real-Time PCR System (Library valid concentration＞10 nM). The clustering of the index-coded samples was performed on a cBot cluster generation system using HiSeq PE Cluster Kit v4-cBot-HS (Illumina) according to the manufacturer’s instructions. After cluster generation, the libraries were sequenced on an Illumina platform and 150 bp paired-end reads were generated.

**Bisulfite sequencing PCR (BSP)**

Cerebral genomic DNA (n = 4 per group) were collected from 2 litters (2 males from each litter) with different fathers. DNA methylation of *Fos* promoter locus was assayed using bisulfite sequencing (Sangon Biotech, Shanghai, China). Genomic DNA was extracted (Ezup column animal genomic DNA purification kit, Sangon, Shanghai, China) and subjected to bisulfite conversion (EZ DNA Methylation-GoldTM Kit D5005, Zymo, CA, USA) according to the manufacturer’s recommendation. Bisulfite-compatible primers (Supplementary Table S7) were used to amplify -1879 to -1505 bp upstream of *Fos* gene transcription start site. PCR products were cloned using pUCm-T vector system (Sangon Biotech, Shanghai, China). 10 individual clones were sequenced per conversion (3730xl DNA Analyzer, Thermo Fisher, MA, USA).
